# Temporary Rise in Blood Thrombogenicity in Patients with Acute Myocardial Infarction

**DOI:** 10.1055/a-1719-6178

**Published:** 2022-01-24

**Authors:** Shumpei Kosugi, Yasunori Ueda, Haruhiko Abe, Kuniyasu Ikeoka, Tsuyoshi Mishima, Tatsuhisa Ozaki, Kohtaro Takayasu, Takuya Ohashi, Haruya Yamane, Masayuki Nakamura, Takashi Fukushima, Kohei Horiuchi, Takashi Iehara, Satoshi Osaki, Kazuki Ozato, Koichi Inoue, Yukihiro Koretsune, Yasushi Matsumura

**Affiliations:** 1 Cardiovascular Division, National Hospital Organization Osaka National Hospital, Osaka, Japan

**Keywords:** blood thrombogenicity, acute myocardial infarction, coronary artery disease, T-TAS

## Abstract

**Objective** Although blood thrombogenicity seems to be one of the determinant factors for the development of acute myocardial infarction (MI), it has not been dealt with in-depth. This study aimed to investigate blood thrombogenicity and its change in acute MI patients.

**Methods and Results** We designed a prospective, observational study that included 51 acute MI patients and 83 stable coronary artery disease (CAD) patients who underwent cardiac catheterization, comparing thrombogenicity of the whole blood between: (1) acute MI patients and stable CAD patients; and (2) acute and chronic phase in MI patients. Blood thrombogenicity was evaluated by the Total Thrombus-Formation Analysis System (T-TAS) using the area under the flow pressure curve (AUC _30_ ) for the AR-chip. Acute MI patients had significantly higher AUC _30_ than stable CAD patients (median [interquartile range], 1,771 [1,585–1,884] vs. 1,677 [1,527–1,756], *p* = 0.010). Multivariate regression analysis identified acute MI with initial TIMI flow grade 0/1 as an independent determinant of high AUC _30_ ( *β* = 0.211, *p* = 0.013). In acute MI patients, AUC _30_ decreased significantly from acute to chronic phase (1,859 [1,550–2,008] to 1,521 [1,328–1,745], *p* = 0.001).

**Conclusion** Blood thrombogenicity was significantly higher in acute MI patients than in stable CAD patients. Acute MI with initial TIMI flow grade 0/1 was significantly associated with high blood thrombogenicity by multivariate analysis. In acute MI patients, blood thrombogenicity was temporarily higher in acute phase than in chronic phase.

## Introduction

Acute myocardial infarction (MI) is caused by plaque disruption and subsequent thrombotic coronary occlusion. However, plaque disruptions and mural thrombus formation are often detected in the coronary arteries without causing any coronary event, that is, silent plaque disruptions. 1 2 The determinant factors for mural thrombus at disrupted plaque to become occlusive have been unknown.

Blood thrombogenicity is one of the well-known determinant factors of thrombus formation. However, it is not always stable, due to which change is likely to influence the development of acute MI. In fact, blood thrombogenicity has been reported to vary under some factors 3 4 5 that may trigger the onset of MI. 6 7 Although a hypothesis that a temporary rise in blood thrombogenicity triggers thrombotic coronary occlusion after plaque disruption has been presented, 8 it appears not to be adequately established. Previous studies investigated thrombogenic components of blood in MI patients, such as platelet function or coagulation factors activities, and suggested that blood thrombogenicity was temporarily increased in the acute phase of MI patients 9 10 11 12 13 14 ; however, these thrombogenic components only partially reflect thrombogenicity of whole blood, which in acute MI patients has not been adequately elucidated.

The present study therefore aimed to test the following pre-specified hypotheses: (1) that thrombogenicity of whole blood is higher in the patients with acute MI than in those with stable coronary artery disease (CAD); and (2) that thrombogenicity of whole blood in MI patients is temporarily high in the acute phase. We used the Total Thrombus-Formation Analysis System (T-TAS; Fujimori Kogyo, Tokyo, Japan) to evaluate thrombogenicity of whole blood under flow conditions. 15

## Material and Methods

### Patients and Study Design

We designed a prospective, single-center, observational study, in which we enrolled patients with acute MI or stable CAD who received left heart catheterization from August 2018 to September 2020, comparing thrombogenicity of the whole blood between 1 acute MI patients and stable CAD patients; and 2 acute and chronic phase in MI patients. Acute MI was defined as type 1 MI according to the fourth universal definition of MI. 16 Stable CAD was defined as asymptomatic or symptomatic CAD with stable symptoms after initial diagnosis or revascularization. Patients using anticoagulants or extracorporeal membrane oxygenation with heparin coating before collection of blood samples were excluded because they were known to affect the measurement by T-TAS. 15 T-TAS measurement was possible only when its specialist was available. This study (EBTAMI [Evaluation of Blood Thrombogenicity in Acute Myocardial Infarction] Study; UMIN000034196) was approved by the Osaka National Hospital Institutional Review Board #2 (Approval No. 16016), and all patients signed written informed consent.

### Antithrombotic Therapy and Catheterization

Antiplatelet therapy was performed with aspirin (100 mg/d) and/or prasugrel (3.75 mg/d) or clopidogrel (75 mg/d) as a maintenance dose for patients who had received percutaneous coronary intervention (PCI). In cases of primary PCI, a loading dose of aspirin (200 mg) and prasugrel (20 mg), or clopidogrel (300 mg) was administered to each naïve patient. Intravenous unfractionated heparin (100 U/kg) was administered at the beginning of catheterization, and an additional dose was repeated to maintain an activated clotting time of ≥250 s during the procedure. GP-IIb/IIIa inhibitors were not used because they were not approved in Japan. Catheterization was performed via radial, brachial, or femoral artery approach using a 6-Fr or 7-Fr sheath and catheters. Coronary angiography was recorded by Artis zee biplane (Siemens Healthineers AG, Erlangen, Germany).

### Collection of Blood Samples

In stable CAD patients and in acute phase MI patients, blood samples were collected before the administration of unfractionated heparin. The administration timing of antiplatelet agents was left to the discretion of attending physician. In MI patients, blood samples were collected again at discharge as the chronic phase. The blood sample was collected into plastic tubes containing 3.2% sodium citrate (Terumo, Tokyo, Japan). It was allowed to stand for 1 to 3 hours, of which 480 µL was mixed with 20 µL of 0.3 mol/L CaCl _2_ containing 1.25 mg/mL of corn trypsin inhibitor immediately before measurement.

### Measurement of Thrombogenicity of Whole Blood

Thrombogenicity of whole blood was determined with T-TAS using the AR chip. The AR chip can assess the thrombogenicity of the whole blood associated with both platelets and coagulation systems. The AR chip contains a capillary channel that is 15 mm long, 300 µm in width, and 80 µm in depth, coated with type I collagen and tissue thromboplastin. The blood sample is perfused through the capillary by the precision pump with a shear rate of 600 s ^−1^ (a flow rate of 10 µL/min). After the perfusion of blood initiated, collagen and tissue thromboplastin activate platelets and the extrinsic coagulation pathway. Depending on the formation of thrombus, the capillary is gradually occluded, and the flow pressure monitored by the sensor is increased. The area under the flow pressure curve for the first 30 minutes at a flow rate of 10 µL/min (AUC _30_ ) was used to evaluate blood thrombogenicity. 15 Prior literatures reported the coefficients of variation for AUC _30_ in AR-chip analysis as 1.2 to 5.0%. 17 18

### Statistical Analysis

Continuous variables were expressed as median (interquartile range [IQR]) and were compared by Mann-Whitney U-test or Kruskal-Wallis test. Categorical variables were expressed as absolute numbers (percentage) and were compared by the Chi-square test or Fisher's exact test. Wilcoxon signed-rank test was used to compare AUC _30_ between acute and chronic phases in MI patients. Spearman rank correlation was used to determine the relationship between AUC _30_ and each variable. Multivariate linear regression analysis was performed to elucidate the factors associated with AUC _30_ , including platelet count, P2Y12 antagonists use, angiotensin-converting enzyme inhibitors/angiotensin II receptor blockers use, beta-blockers use, and acute MI (model 1) or acute MI with TIMI flow grade 0/1 (model 2). Variables with *p* <0.10 by univariate analysis were used for these models. All statistical analysis was regarded as significant when *p* -value was <0.05. Statistical analysis was performed by IBM SPSS Statistics 23.0 software (IBM Corp., Armonk, New York, United States).

## Results

### Study Patients

Included in the analysis were 51 patients with acute MI and 83 patients with stable CAD. Patients' characteristics are presented in Table 1 . Platelet count was higher in the acute MI patients than in stable CAD patients, whereas antiplatelet agents were used more frequently in stable CAD patients than in acute MI patients. There was no significant difference in AUC _30_ among patients taking no, single, and dual antiplatelet agents both in acute MI patients (1,794 [1,598–1,908] vs. 1,748 [1,560–1,876] vs. 1,752 [1,255–1,853], *p* = 0.546) and in stable CAD patients (1,593 [1,538–1,651] vs. 1,727 [1,494–1,774] vs. 1,681 [1,552–1,775], *p* = 0.436).

**Table 1:** Baseline characteristics of patients with acute MI or stable CAD

Variables	Acute MI	Stable CAD	*p* -Value
Study patients	51	83	–
Age, years	70 (60–79)	71 (66–77)	0.615
Male sex	38 (75)	66 (80)	0.499
Body-mass index, kg/m ^2^	22.6 (20.3–25.0)	24.1 (21.7–26.8)	0.011
STEMI	27 (53)	–	–
Initial TIMI flow grade 0/1	22 (43)	–	–
Prior MI	4 (8)	20 (24)	0.017
Prior PCI	10 (20)	50 (60)	<0.001
Coronary risk factors			
Hypertension	29 (57)	58 (70)	0.125
Hypercholesterolemia	28 (55)	55 (66)	0.188
Diabetes mellitus	19 (37)	34 (41)	0.670
Current smoker	29 (57)	25 (30)	0.002
Medical treatment			
Aspirin	35 (69)	77 (93)	<0.001
P2Y12 antagonists	8 (16)	70 (84)	<0.001
Beta-blockers	4 (8)	34 (41)	<0.001
ACE-inhibitors/ARB	14 (27)	51 (61)	<0.001
Calcium channel antagonists	14 (27)	37 (45)	0.047
Statins	17 (33)	68 (82)	<0.001
Oral hypoglycemic drug	11 (22)	15 (18)	0.619
Laboratory data			
Platelet count, 10 ^3^ /µL	225 (191–295)	203 (169–231)	0.002
Hematocrit, %	40.8 (36.3–44.7)	39.9 (37.0–42.5)	0.318
CRP, mg/dL	0.12 (0.05–0.53)	0.09 (0.03–0.18)	0.022
Total cholesterol, mg/dL	187 (141–227)	157 (135–173)	<0.001
LDL cholesterol, mg/dL	108 (87–143)	79 (66–94)	<0.001
HDL cholesterol, mg/dL	48 (40–56)	49 (43–60)	0.381
Triglycerides, mg/dL	91 (57–161)	119 (89–154)	0.027
Creatinine, mg/dL	0.95 (0.81–1.22)	0.87 (0.74–1.04)	0.083
HbA _1c_ , %	6.0 (5.6–6.7)	6.1 (5.8–6.9)	0.203
CK, U/L	92 (66–208)	104 (67–154)	0.576
CK-MB, U/L	5 (4–24)	–	–
Troponin T, ng/L	123 (40–522)	–	–

Abbreviations: ACE, angiotensin-converting-enzyme; ARB, angiotensin II receptor blockers; CAD, coronary artery disease; CK, creatine kinase; CRP, C-reactive protein; HbA _1c_ , glycosylated hemoglobin; HDL, high-density lipoprotein; LDL, low-density lipoprotein; MI, myocardial infarction; PCI, percutaneous coronary intervention; STEMI, ST-segment elevation myocardial infarction.

Note: Categorical variables are described as absolute numbers (%) and continuous variables are described as median (interquartile range).

### Comparison between Patients with Acute MI and Stable CAD

**Fig. 1:**
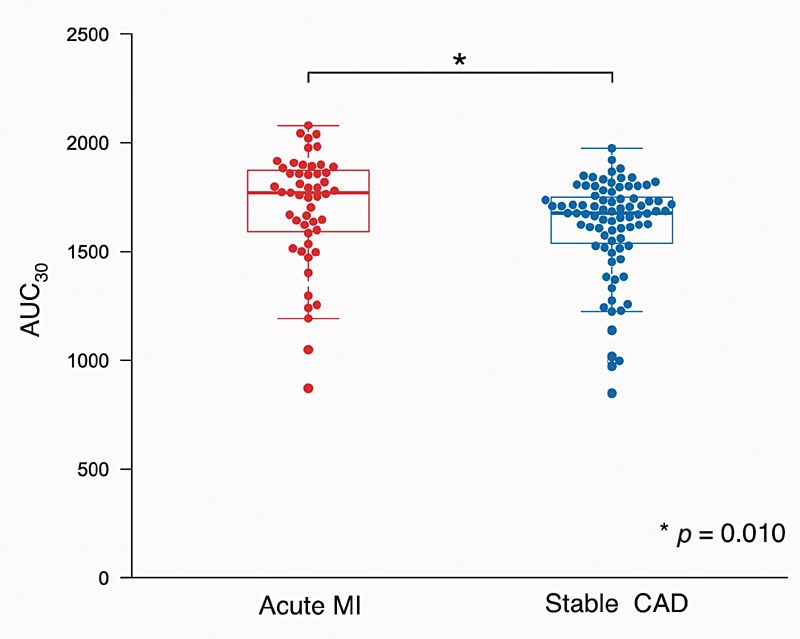
*Comparison of blood thrombogenicity between acute MI and stable CAD.* Patients with acute MI had significantly higher AUC _30_ levels than those with stable CAD (1,771 [1,585–1,884] vs. 1,677 [1,527–1,756], *p* = 0.010). CAD, coronary artery disease; MI, myocardial infarction.

**Fig. 2:**
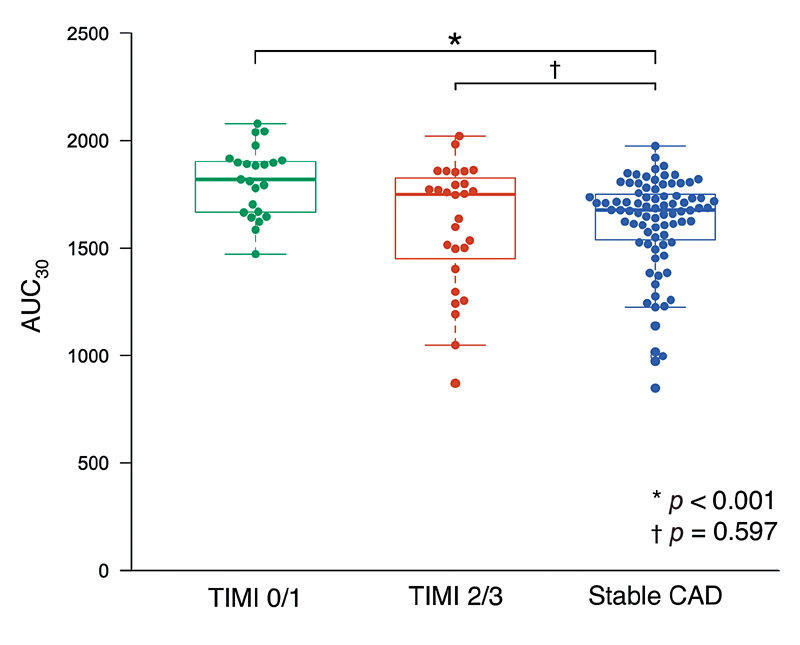
*Comparison of blood thrombogenicity by initial TIMI flow grade in acute MI.* Acute MI patients with initial TIMI 0/1 flow grade had significantly higher AUC _30_ levels than stable CAD patients (1,852 [1,661–1,910] vs. 1,677 [1,527–1,756], *p* <0.001), whereas there was no significant difference in AUC _30_ levels between acute MI patients with initial TIMI 2/3 flow grade and stable CAD patients (1,748 [1,450–1,826] vs. 1,677 [1,527–1,756], *p* = 0.597). CAD, coronary artery disease; MI, myocardial infarction.

Acute MI patients had significantly higher AUC _30_ than stable CAD patients (1,771 [1,585–1,884] vs. 1,677 [1,527–1,756], *p* = 0.010; Fig. 1 ). Acute MI patients with initial TIMI flow grade 0/1 had significantly higher AUC _30_ than stable CAD patients, whereas there was no significant difference in AUC _30_ between acute MI patients with initial TIMI flow grade 2/3 and stable CAD patients (1,852 [1,661–1,910] vs. 1,677 [1,527–1,756], *p* < 0.001; 1,748 [1,450–1,826] vs. 1,677 [1,527–1,756], *p* = 0.597, respectively; Fig. 2 ). Multivariate linear regression analysis ( Table 2 ) revealed that an independent determinant of high blood thrombogenicity was acute MI with TIMI flow grade 0/1.

**Table 2:** Multivariate liner regression analysis of determinants of AUC _30_ levels

Variables	*β*	95% CI	*p* -Value
Model 1			
Platelet count	0.367	0.207–0.527	<0.001
Model 2			
Acute MI with TIMI 0/1 flow grade	0.211	0.044–0.377	0.013
Platelet count	0.297	0.131–0.464	0.001

Abbreviations: CI, confidence interval; MI, myocardial infarction.

Among 24 non-ST-segment elevation MI patients, six patients had multiple stenotic lesions that might be the possible culprit of MI, in whom the culprit of MI was determined by echocardiographic findings in three patients, by electrocardiographic findings in two patients, and by the flow delay on angiogram in one patient. On the other hand, the rest 18 patients had only one stenotic lesion that can be the culprit of MI. Since the identification of MI culprit might be difficult in some patients with non-ST-segment elevation MI, the additional analysis regarding TIMI flow grade was performed with ST-segment elevation MI patients alone. The ST-segment elevation MI patients with TIMI 0/1 ( *n* = 21) had significantly higher AUC _30_ than stable CAD patients, whereas there was no significant difference in AUC _30_ between ST-segment elevation MI patients with TIMI 2/3 ( *n* = 6) and stable CAD patients (1,819 [1,656–1,912] vs. 1,677 [1,527–1,756], *p* <0.001; 1,636 [1,437–1,810] vs. 1,677 [1,527–1,756], *p* = 1.000, respectively). Furthermore, multivariate analysis showed that ST-segment elevation MI with TIMI flow grade 0/1 was an independent determinant of high AUC _30_ ( *β* = 0.193, *p* = 0.024). These results were not different from the analysis with all MI patients.

### Comparison between the Acute and Chronic Phases in Acute MI Patients

**Fig. 3:**
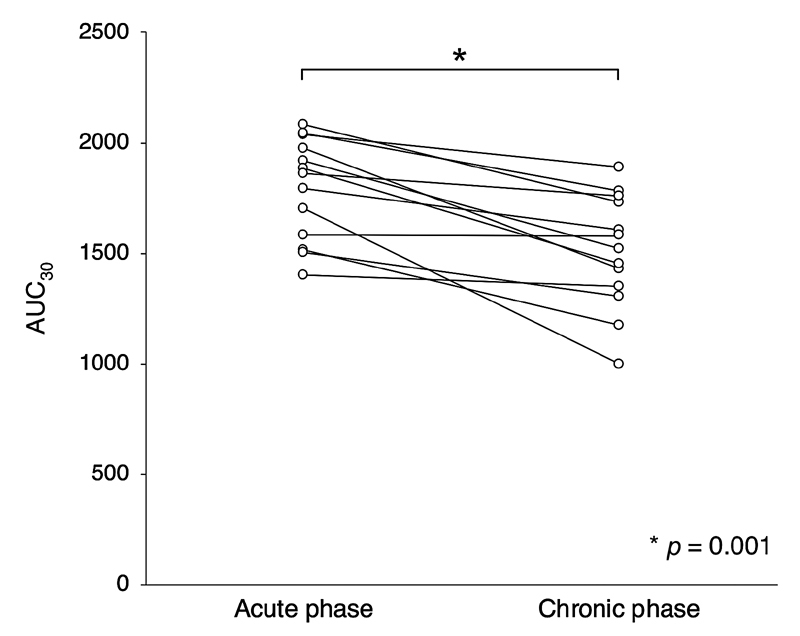
*Serial changes of blood thrombogenicity from the acute to chronic phase in acute MI.* AUC _30_ levels decreased significantly from acute to chronic phase in patients with acute MI (1,859 [1,550–2,008] to 1,521 [1,328–1,745], *p* = 0.001). MI, myocardial infarction.

Both of acute phase and chronic phase T-TAS measurements were available in 13 patients. Baseline characteristics are presented in Table 3 . The measurement interval between acute and chronic phase was 13 ± 5 days. AUC _30_ decreased significantly from acute to chronic phase in those patients (1,859 [1,550–2,008] to 1,521 [1,328–1,745], *p* = 0.001; Fig. 3 ).

**Table 3:** Baseline characteristics of patients with acute MI in the acute or chronic phase

Variables	Acute phase	Chronic phase	*p* -Value
Medical treatment			
Aspirin	9 (69)	13 (100)	0.125
P2Y12 antagonists	0 (0)	13 (100)	<0.001
Beta-blockers	1 (8)	12 (92)	0.001
ACE-inhibitors/ARB	2 (15)	11 (85)	0.004
Calcium channel antagonists	2 (15)	0 (0)	0.500
Statins	3 (23)	13 (100)	0.002
Laboratory data			
Platelet count, 10 ^3^ /µL	240 (191–315)	237 (212–302)	0.724
Hematocrit, %	40.7 (38.7–46.0)	38.0 (35.6–43.0)	0.009
CRP, mg/dL	0.18 (0.08–0.30)	0.37 (0.17–1.39)	0.060
Total cholesterol, mg/dL	204 (147–229)	142 (131–170)	0.001
LDL cholesterol, mg/dL	136 (96–153)	80 (71–103)	0.001
HDL cholesterol, mg/dL	47 (39–52)	41 (35–65)	0.003
Triglycerides, mg/dL	117 (43–154)	123 (89–134)	0.807
Creatinine, mg/dL	0.89 (0.72–1.21)	0.85 (0.75–1.07)	0.421
CK, U/L	80 (68–141)	47 (35–65)	0.001

Abbreviations: ACE, angiotensin-converting-enzyme; ARB, angiotensin II receptor blockers; CK, creatine kinase; CRP, C-reactive protein; HDL, high-density lipoprotein; LDL, low-density lipoprotein; MI, myocardial infarction.

Note: Categorical variables are described as absolute numbers (%) and continuous variables are described as median (interquartile range).

## Discussion

The present study demonstrated that thrombogenicity of whole blood was significantly higher in acute MI patients, especially in those with TIMI flow grade 0/1, than in stable CAD patients. Furthermore, blood thrombogenicity decreased significantly from acute to chronic phase among acute MI patients, suggesting that thrombogenicity was temporarily high in the acute phase.

Several studies have demonstrated the association between the onset of MI and coagulation factors. The increased levels of fibrinogen and von Willebrand factor antigen were independent predictors of subsequent acute coronary syndromes in a prospective cohort study with angina pectoris patients. 9 The increased factor VII, IX, and XI activity, and the decreased levels of antithrombin III and protein C were detected in the acute phase of MI patients. 10 11 12 Moreover, the activation of factor XI and increased serum levels of fibrinopeptide A were temporarily observed in the acute phase of MI patients. 13 14 On the other hand, factor X did not differ between acute MI and stable CAD patients, 11 and factor V and XII were rather reduced in the acute phase of MI patients. 10 12 Therefore, in those prior studies, thrombogenicity of whole blood cannot be judged from the findings on the individual coagulation factors. The importance and novelty of our study are that whole blood samples under flow conditions mimicking in vivo atherosclerotic lesion were used to investigate the status of blood thrombogenicity in acute MI patients. The present study demonstrated that blood thrombogenicity is temporarily high in the acute phase of MI patients, supporting the hypothesis that a temporary increase in blood thrombogenicity is involved in the development of acute MI.

We found that MI patients with initial TIMI flow grade 0/1 was independently associated with high AUC _30_ . This finding may be pathophysiologically plausible. High blood thrombogenicity may cause occlusive large and firm thrombus formation leading to TIMI flow grade 0/1; however, relatively low blood thrombogenicity may cause the formation of relatively fragile thrombus leading to spontaneous recanalization with the higher TIMI flow grade, which results in the stronger relationship between AUC _30_ and TIMI 0/1 acute MI rather than with acute MI in general.

The present study showed that platelet count was also independently associated with AUC _30_ , which was consistent with previous studies. 19 20 It is controversial whether platelet count is a risk of MI, as some population-based cohort studies have reported high platelet count as a risk of cardiovascular death and cardiovascular disease 21 22 but others have reported that it is not. 23 24 However, since acute MI patients had higher platelet count than stable CAD patients and platelet count was independently associated with high blood thrombogenicity in the present study, platelet count would be a risk of MI.

The measurement of blood thrombogenicity by T-TAS using AR chip is known to be affected by anticoagulants, GPIIb/IIIa antagonists, and GPIbα antagonists 15 20 but not by aspirin and P2Y12 antagonists, i.e., clopidogrel, prasugrel, and ticagrelor. 17 19 25 26 The reason for this has been mentioned in prior literature that P2Y12 inhibitors may have no effect in AR chip, in which thrombus formation is more dependent upon fibrin formation, as platelets play little in thrombus formation at low shear rates. 17 AR chip measures the formation of occlusive thrombus in a capillary channel coated with type I collagen and tissue thromboplastin, mimicking the formation of occlusive thrombus in the atherosclerotic artery. Aspirin and P2Y12 inhibitors have an antithrombotic effect through GPIIb/IIIa, which mainly contributes to platelet instability. 27 Although platelets are generally important for the arterial thrombus formation, thrombus formed at the culprit lesion of MI is mixed thrombus with high fibrin content, 28 29 suggesting that coagulation system plays an important role in the formation of occlusive thrombus. We therefore believe that the measurement using T-TAS with AR-chip would be appropriate for assessing blood thrombogenicity related to the development of acute MI.

Epidemiologic studies have shown that various factors such as temperature, exercise, and mental stress trigger the onset of MI 6 7 and that those factors increase platelet and coagulation activities. 3 4 5 Our present study added important evidence for the association between the increase in blood thrombogenicity and acute MI onset. However, further studies are still needed to clarify the mechanisms that contribute to the formation of occlusive thrombus at the thrombogenic vessel wall by which acute MI is caused. The hypothesis 30 that a temporary increase in blood thrombogenicity is involved in the development of acute MI should also be tested in those studies.

Although AR-chip mimics the formation of occlusive thrombus in the atherosclerotic artery, nobody knows if it is the ideal model for acute MI culprit lesion. The dynamic circadian, daily, or monthly changes of blood thrombogenicity and its determinant factors has not yet been clarified.

## Conclusion

Blood thrombogenicity was significantly higher in acute MI patients than in stable CAD patients. Acute MI with initial TIMI flow grade 0/1 was significantly and independently associated with high blood thrombogenicity by multivariate analysis. Furthermore, in acute MI patients, blood thrombogenicity was temporarily higher in acute phase than in chronic phase.

## Notes

**What Is Known about This Topic?**

- Blood thrombogenicity seems to be one of the determinant factors for mural thrombus at disrupted coronary plaques to become occlusive.
- Acute myocardial infarction is associated with a hypercoagulable state with increased fibrinogen, von Willebrand factor antigen, and factor VII, IX, and XI activity, and decreased antithrombin III and protein C.

**What Does This Paper Add?**

- Thrombogenicity of whole blood measured by T-TAS was significantly higher in the patients with acute myocardial infarction than in those with stable coronary artery disease.
- In acute myocardial infarction patients, thrombogenicity of the whole blood was temporarily higher in acute phase than in chronic phase.
